# Role of concentric needle Single Fiber Electromyography in detection of subclinical motor involvement in carpal tunnel syndrome

**DOI:** 10.1186/s41983-018-0004-4

**Published:** 2018-04-25

**Authors:** Aliaa A. Tawfeek, Amani M. Nawito, Radwa M. Azmy, Amr Hassan, Lamia M. Afifi, Saly H. Elkholy

**Affiliations:** 1 Clinical Neurophysiology Unit, Kasr Alainy Hospital, Faculty of Medicine, Cairo University, Cairo, Egypt; 2 Neurology department, Kasr Alainy Hospital, Faculty of Medicine, Cairo University, Cairo, Egypt

**Keywords:** Single-fiber electromyography, Concentric needle, Mean consecutive difference, Carpal tunnel syndrome, Abductor pollicis brevis

## Abstract

**Background:**

Conventional motor nerve conduction studies are usually normal in early and mild carpal tunnel syndrome (CTS). Single-fiber electromyography (SFEMG) measures the mean consecutive difference (MCD) as an expression of the variability in impulse transmission over the motor endplates and along the nerve fibers distally to the last branching point and along the muscle fibers.

Application of concentric needle SFEMG in a group of CTS patients who showed pure sensory abnormalities in nerve conduction studies to examine for subclinical motor involvement.

**Methods:**

Thirty CTS patients having only sensory involvement proved clinically and by conventional electrophysiological studies were included in addition to 30 control subjects. Concentric needle SFEMG was performed to the abductor pollicis brevis (APB), abductor digiti minimi (ADM), and extensor digitorum communis (EDC) muscles.

**Results:**

There was a statistically significant difference in the MCD between the patient and control groups in the APB only (*p* = 0.038).

**Conclusions:**

The results suggest the presence of a subclinical motor median neuropathy at the wrist in patients with early and mild carpal tunnel syndrome and highlight the validity of the concentric needle SFEMG in early neuropathies.

**Trial registration:**

PACTR201802002971380 registered 12 February 2018, retrospectively registered.

## Background

Conventional electrophysiological studies in early and mild carpal tunnel syndrome (CTS) often show mild median sensory nerve conduction abnormalities across the wrist leading to the widely accepted assumption that sensory abnormalities precede motor abnormalities (Bland 2000). The median sensory conduction studies are thus considered more sensitive than motor conduction studies in the electrodiagnosis of CTS (Jablecki et al. 1993).

However, normal motor conduction studies in CTS may be attributed to a lower sensitivity of the conventional wrist to abductor pollicis brevis (APB) motor study method to detect abnormalities, rather than sparing of the motor fibers, especially in mild CTS patients (Chang et al. 2002, 2006, 2009; Ginanneschi et al. 2006).

Single-fiber electromyography (SFEMG) measures the variation in the time interval between the two muscle action potentials supplied by the same axon over the motor endplates, also along the nerve fibers distally to the last branching point and along the muscle fibers under investigation (Thiele and Stålberg 1975).

SFEMG is used mainly as a sensitive diagnostic method of myasthenia gravis and other disorders with disturbed neuromuscular transmission. It also contributes to the understanding of reinnervation dynamics and has found a place in the neurogenic diseases and myopathies (Sanders and Stålberg 1996).

Due to the cost of the single-fiber electrode, the use of the SFEMG technique is limited in many labs. Introducing the concentric needle electrode opened a fruitful optimizing window to apply this sophisticated technique, which was validated in many studies (Ertas et al. 2000; Benatar et al. 2006; Sarrigiannis et al. 2006; Kouyoumdjian and Stalberg 2008; Stålberg and Sanders 2009).

This study aimed at applying concentric needle SFEMG in a group of CTS patients in whom only sensory abnormalities of the median nerve were established by conventional nerve conduction studies, to examine if there is a subclinical motor involvement. This may add to the current understanding of the effects of median nerve compression in early and mild stages which may have an impact on the choice of treatment regimen, and ultimately improve the prognosis.

## Methods

The study included 60 subjects, 57 females and 3 males. They were divided into two groups: a patient group and a control group. The patient group included 29 females and 1 male patient. Their ages ranged from 25 to 53 years, with a mean age of 35.4 ± 7.8 years. Patients included in the study were clinically suspected to have carpal tunnel syndrome, and their sensory nerve conduction studies showed early starting or mild degrees of entrapment according to the classification of Bland (2000) (The mild degree of entrapment corresponds to delayed median nerve sensory peak latency and normal motor studies, while in early starting entrapment there is normal sensorimotor median nerve conduction studies and positive comparative studies). We used the median-ulnar ring finger antidromic comparative sensory studies (American Association of Electrodiagnostic Medicine et al. 2002). We excluded patients with other neuromuscular disorders, proven clinically or electrodiagnostically, e.g., cervical radiculopathy and polyneuropathy. We also excluded patients with systemic diseases which may be associated with neuropathy, e.g., diabetes mellitus, and patients in whom EMG examination is contraindicated, e.g., bleeding disorders. We also recruited 30 age- and sex-matched healthy volunteers to serve as a control group; their ages ranged from 22 to 43 years with mean age of 32.7 ± 5.9 years.

### Clinical assessment

Full history taking and thorough clinical examination were undertaken with highlights on decreased overall sensation, response to pinprick and light touch over the palmar surface of the lateral three and half fingers, and thenar weakness. Tinel sign, Phalen test, and median nerve compression test were performed.

### Electrodiagnosis

Electrophysiologic studies were carried out using a Nihon Kohden® MEB_9200K Neuropack machine (Tokyo, Japan), software V.08.11 (Tokyo, Japan), in the Clinical Neurophysiology unit of Kasr Alainy Hospital, Cairo University. Motor and sensory nerve conduction studies (NCS) to the median and ulnar nerves and median-ulnar ring finger antidromic sensory studies were performed according to standard techniques (Preston and Shapiro 2013). The normal cut off values were as follows: *Median motor NCS*: distal latency ≤ 4.4 ms, amplitude ≥ 4.0 mV, and conduction velocity ≥ 49 m/s. *Ulnar motor NCS*: distal latency ≤ 3.3 ms, amplitude ≥ 6.0 mV, and conduction velocity ≥ 49 m/s. *Median sensory NCS*,: peak latency ≤ 3.5 ms, amplitude ≥ 20 μV, conduction velocity ≥ 50 m/s. *Ulnar sensory NCS*: peak latency ≤ 3.1 ms, amplitude ≥ 17 μV conduction velocity ≥ 50 m/s. *Median-ulnar ring finger antidromic sensory study* was considered normal up to 0.4 ms peak latency difference (Preston and Shapiro 2013).

EMG examination and SFEMG were carried out using a disposable small concentric needle with a recording area of 0.031 mm^2^ (Technomed®, Netherland). The muscles examined for patients and controls were the abductor pollicis brevis (APB), abductor digiti minimi (ADM), and extensor digitorum communis (EDC) muscles. For conventional EMG, the pattern of insertional activity, the presence or absence of resting activity, the motor unit potentials morphology at moderate contraction, the pattern of recruitment, and the interference pattern at maximum effort were assessed. For SFEMG, the high- and low-frequency filters were 10 and 2 kHz, respectively (Ertas et al. 2000). The needle was inserted in the muscle and the subject was instructed to perform weak contraction of the examined muscle. The needle was manipulated carefully until a stable signal of at least two upward peaks was shown on the monitor. Then, the needle was kept in position until 50–100 sweeps of the fiber pair were recorded. The position of the needle was then changed and the procedure was repeated 10 times to collect 10 fiber pairs for each subject with a total of 300 fiber pairs for each group. For each fiber pair, the mean consecutive difference (MCD) was calculated as well as the mean MCD of all trials and if there was abnormal blocking. When the mean consecutive difference/mean sorted difference (MCD/MSD) value exceeded 1.25, which means the inter-potential interval was influenced by the variations in the firing rate, we used the MSD instead of the MCD to represent the neuromuscular jitter, because the MSD is a mathematical algorithm that reduces the effect of this factor (Sanders and Stålberg 1996).

### Statistical methods

Patients’ data were tabulated and processed using software Microsoft Excel 2010. Quantitative variables were expressed by mean ± standard deviation (SD). Independent samples *t*-test was used to assess the significance of differences between two subject groups, and *p* value ≤ 0.05 was considered statistically significant.

## Results

### Clinical characteristics

As regards symptomatology, the main complaint of the patients was tingling and numbness of the affected hand and 50% of the patients experienced nocturnal pain and parasthesias in the affected limb that awakened them from sleep, but none had thenar weakness. On examination, only three patients (10%) showed decreased sensation over the digits supplied by the median nerve by pin prick examination. Phalen test was positive in 12 (40%) patients. Tinel sign was positive in 8 (26.7%) patients. Direct compression of the median nerve elicited pain in 4 (13.3%) patients.

### Electrophysiologic results

The median sensory conduction data of the patient group are summarized in Table 1. According to these data, the patient group was sub-classified into: 16 patients (53.3%) who had early starting degree of entrapment, while 14 patients (46.7%) had mild degree of entrapment. Conventional EMG showed normal motor unit potentials morphology and recruitment of all examined muscles in both groups.

**Table 1:** Values of median sensory conduction studies of the patient group

Sensory conduction studies	Minimum	Maximum	Mean ± SD
Median onset latency(ms)	2.6	3.8	2.9 ± 0.3
Median peak latency(ms)	3.1	4.5	3.7 ± 0.4
Median amplitude (μV)	7.0	69.1	32.1 ± 16.9
Median distal CV	35.0	50.8	44.3 ± 4.3
Median vs. ulnar 4th digit comparative sensory study	0.5	2.3	1.2 ± 0.5

*CV* conduction velocity

**Fig. 1:**
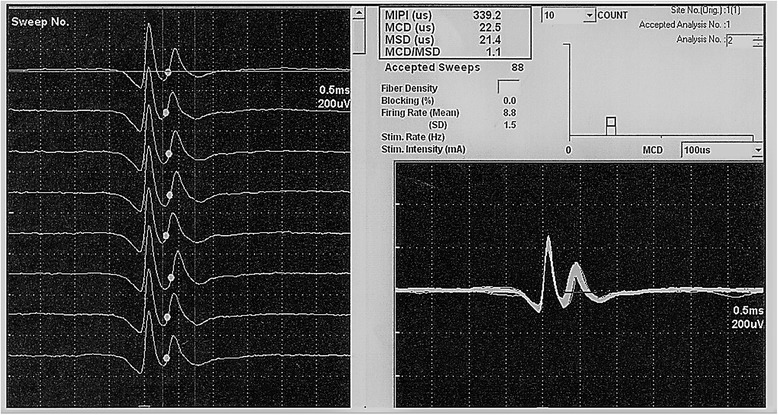
SFEMG potentials recorded from APB during voluntary contraction. Trace obtained from a control subject. MCD is 22.5 μs

**Fig. 2:**
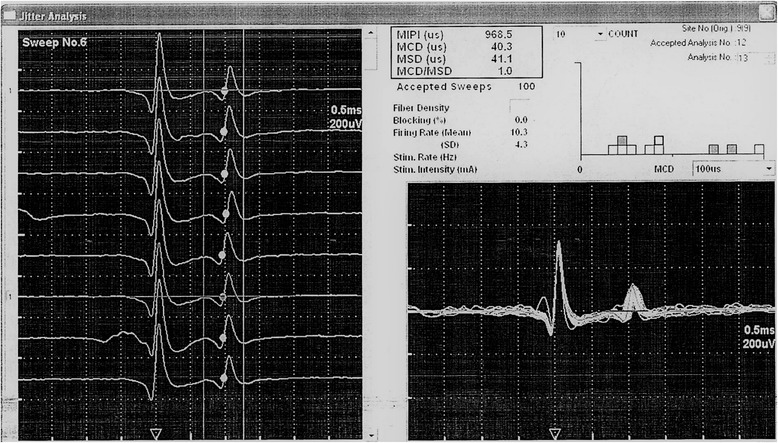
SFEMG potentials recorded from APB during voluntary contraction. Trace obtained from a CTS patient. MCD is = 40.3 μs

On comparing the results of SFEMG in both groups, we found statistically significant higher MCD values of APB in the patient group as compared to the control group (Figs. 1 and 2). However, no statistically significant difference was found in MCD values of EDC and ADM (Table 2).

**Table 2:** MCD of the APB, EDC, and ADM

Muscle	APB		EDC		ADM	
	Patients (300 fiber pairs)	Control (300 fiber pairs)	Patients (300 fiber pairs)	Control (300 fiber pairs)	Patients (300 fiber pairs)	Control (300 fiber pairs)
*MCD*						
*Mean* ± *SD*	25.00 ± 9.1	23.56 ± 7.88	24.58 ± 8.73	25.71 ± 9.00	23.37 ± 8.1	24.12 ± 7.78
*p value*	0.038*		0.12		0.25	

*ABD* abductor pollicis brevis, *EDC* extensor digitorum communis, *ADM* abductor digiti minimi, *MCD* mean consecutive difference

*p value < 0.05 = significant*

Comparison of MCD of the APB muscle between the mild CTS group and early starting one (25.65 ± 9.14 μs with 140 fiber pairs, 24.44 ± 9.06 μs with 160 fiber pairs respectively) did not show a statistically significant difference (*p* = 0.25).

## Discussion

This study included a group of CTS patients with only sensory involvement proved by conventional nerve conduction studies. Our aim was to search for an undetected motor affection using concentric needle SFEMG. The importance of this work rests on the fact that electrodiagnosis is usually required before consideration of local pharmacological treatment or surgery.

Clinically, while all of our patients showed sensory symptoms suggestive of CTS, on examination, decreased sensation over the fingers supplied by the median nerve was detected in only 10% of patients. Kuhlman and Hennessey (1997) found decreased sensation positive in 51%. This variability may be due to the difference in the degree of compression of the median nerve in the samples studied. Our patients showed also no motor signs or symptoms which conforms to the published data that in early stages of CTS, no weakness is expected (MacDermid and Wessel 2004).

Phalen, Tinel, and median nerve compression tests were positive in 40, 26.7, and 13.3% respectively which were less than those of other studies (Kuhlman and Hennessey 1997; MacDermid and Wessel 2004; Wiesman et al. 2003). Most likely, this was because we included patients with early and mild stages of CTS.

As regards the SFEMG, it was carried out using a small concentric needle to the APB, EDC, and ADM, since SFEMG abnormalities are not specific to a particular etiology (Benatar et al. 2006). The mean MCD between the patient and control groups of the three muscles showed a statistically significant difference in the APB muscle only, while those of the ADM and EDC muscles were insignificant, which localizes the abnormality to the APB muscle. This is in agreement with the studies that show evidence of the lower sensitivity of standard motor electrodiagnostic techniques to detect abnormalities, rather than sparing of motor axons (Chang et al. 2002, 2006, 2009; Ginanneschi et al. 2006).

To the best of our knowledge, there is only one previous study that assessed CTS using the SFEMG (stimulated technique) (Farouk 2006) with a single-fiber needle and it showed prolonged MCD of the APB. A study using SFEMG in acute stages of Guillian-Barre syndrome showed increased MCD at varying degrees in the tested patients (Spaans et al. 2003). Similarly, a SFEMG study in chronic inflammatory demyelinating polyneuropathy showed minimally abnormal jitter in 74% of their cases (Oh 1989). Another study used SFEMG in polyneuropathies of different etiologies (demyelinating and axonal) and found different degrees of increased MCD, in the studied subgroups (Thiele and Stålberg 1975).

Abnormal blocking was not detected in our patients. There are several possible reasons for this: first, the fact that our patients were in early and mild stages of CTS. Second, the concentric needle has a larger recording area than the SFEMG needle, thus making it more likely to produce recordings with overlapping pairs caused by contributions from more distant motor units within the recording area. Last, it could be an indication that the form of neuropathy is a demyelinating one, which on the one hand is in accordance with Thiele and Stålberg (1975) who did not find abnormal blocking in demyelinating neuropathy and Oh (1989) who found only rare blocking; on the other hand, it does not match with Spaans et al. (2003) who found abnormal blocking ranging from 10 to 32% of muscle fiber action potentials and Farouk (2006), who found abnormal blocking in 36.7% of cases.

The fiber density as a measure could not be taken into consideration in our study due to the use of the concentric needle because the recorded spikes are often a composite of the activities of more than one muscle fiber.

Comparison of MCD of the APB muscle of the mild CTS group and early starting one was not statistically significant, which may be because the difference in the degree of entrapment is minimal.

## Conclusions

We conclude that SFEMG may be a useful tool in detecting the abnormalities affecting the motor branches of the APB which are missed by conventional nerve conduction techniques at the early and mild stages of carpal tunnel syndrome. These abnormalities highlight also that concentric needle SFEMG may be a useful technique in detecting early neuropathies despite that the concentric needle has a larger recording area, which might tend to underestimate the true jitter value; however, further studies are needed in that field.

## Abbreviations

ADM: Abductor digiti minimi
APB: Abductor pollicis brevis
CTS: Carpal tunnel syndrome
CV: Conduction velocity
EDC: Extensor digitorum communis
EMG: Electromyography
MCD: Mean consecutive difference
NCS: Nerve conduction studies
SFEMG: Single-fiber electromyography
